# The underlying molecular mechanisms and biomarkers of plaque vulnerability based on bioinformatics analysis

**DOI:** 10.1186/s40001-022-00840-7

**Published:** 2022-10-27

**Authors:** Rui Cheng, Xiaojiang Xu, Shurong Yang, Zhongqian mi, Yong Zhao, Jinhua gao, Feiyan Yu, Xiuyun Ren

**Affiliations:** 1 Shanxi Medical University, 56 Xinjian South Road, Taiyuan, 030001 Shanxi China; 2 Department of Endocrinology, the Second Hospital of Shanxi Medical University, 382 Wuyi Road, Taiyuan, 030001 Shanxi China; 3 Shanxi Medical University School and Hospital of Stomatology, 63# Xinjian South Road, Taiyuan, 030001 Shanxi People’s Republic of China; 4 Shanxi Province Key Laboratory of Oral Diseases Prevention and New Materials, Taiyuan, 030001 China

**Keywords:** Atherosclerosis, Differentially expressed genes, Macrophages, Unstable atherosclerotic plaque, Transcription factors

## Abstract

**Aim:**

The study aimed to identify the underlying differentially expressed genes (DEGs) and mechanism of unstable atherosclerotic plaque using bioinformatics methods.

**Methods:**

GSE120521, which includes four unstable samples and four stable atherosclerotic samples, was downloaded from the GEO database. DEGs were identified using LIMMA. Gene Ontology (GO) and Kyoto Encyclopedia of Genes and Genomes (KEGG) enrichment analyses of DEGs were performed using the Database for metascape Visualization online tool. Based on the STRING database, protein–protein interactions (PPIs) network among DEGs were constructed. Regulatory networks were visualized using Cytoscape. We use the xCell to analyze the different immune cell subtypes.

**Results:**

A total of 1626 DEGs (1034 up-regulated and 592 down-regulated DEGs) were identified between unstable and stable samples. I pulled 62 transcription factors (34 up-regulated TFs and 28 down-regulated TFs) from the Trust database. The up-regulated TFs were mainly enrichment in positive regulation of myeloid leukocyte differentiation, and the down-regulated TFs were mainly enrichment in connective tissue development. In the PPI network, RB1, CEBPA, PPARG, BATF was the most significantly up-regulated gene in ruptured atherosclerotic samples. The immune cell composition enriched in CD cells and macrophages in the unstable carotid plaque.

**Conclusions:**

Upregulated RB1, CEBPA, PPARG, BATF and down-regulated SRF, MYOCD, HEY2, GATA6 might perform critical promotional roles in atherosclerotic plaque rupture, furthermore, number and polarization of macrophages may play an important role in vulnerable plaques.

**Supplementary Information:**

The online version contains supplementary material available at 10.1186/s40001-022-00840-7.

## Introduction

Cardiovascular and cerebrovascular diseases are the main causes of human death, in which AS is the culprit. Atherosclerosis (AS) is a chronic inflammatory disease which is characterized by the formation of medium- and large-sized atherosclerotic plaques on the vascular walls [1]. Moreover, of all atherosclerotic, carotid AS accounts for about 25%, easier to occur at the bifurcation of the carotid artery and is strongly associated with stroke [2]. Vulnerable plaque rupture, secondary thrombosis or embolism are major causes of stroke [3]. Pathology studies showed that vulnerable atherosclerotic plaques have a obvious morphology of thin-cap fibroatheromas, covering a large lipidic and necrotic core [4].

Activation of the immune system promotes the biological processes of vulnerable plaques [5]. Differences in cellular composition between vulnerable and stable plaques have been well established [6]. Macrophages are key players in cardiovascular disease (CVD) and have an important influence on the stability of atherosclerotic plaques.

In recent decades, the study found that transcription factors are key cellular components that control gene expression, many of them as “master regulators” exerting control over processes that specify cell types and developmental patterning and controlling specific pathways such as immune response [7]. For example, IRF5 is a major regulator of macrophage activation, regulates macrophage phenotypes and co-locates with CD11c + macrophages on the shoulder of plaques. It is associated with plaque vulnerability and symptoms of carotid endarterectomy in humans. In a mouse model of induced carotid artery plaque rupture, IRF5 drives plaque rupture [8]. In addition, researchers found that macrophage NCOR1 could block the pro-atherogenic effect of PPARc and suggest that stabilizing the NCOR1–PPARc binding could be a promising strategy to block the pro-atherogenic functions of plaque macrophages and lesion progression in atherosclerotic patients. Therefore, NCOR1 plays an important role in the regulation of atherosclerosis [9]. However, specific regulation mechanisms of transcription factors (TFs) and miRNAs involved in the progression of plaque stability have not been revealed.

In our study, we analyzed the regulatory role of a group of TFs in atherosclerotic plaques. The database obtained from GEO, we further explored the potential biological process of the dysregulated TFs and mRNAs relying on a variety of biological tools. Finally, we constructed the TFs–mRNA network and screened for potential hub genes to provide an important target for preventing the progression of atherosclerotic plaque.

## Materials and methods

### Subjects

#### Bioinformatics datasets

Expression profiling by high-throughput sequencing of mRNA GSE120521 was obtained from the platform Illumina HiSeq 2500 (Homo sapiens) of Gene Expression Omnibus database (GEO, https://www.ncbi.nlm.nih.gov/geo/).

## Data preprocessing and identification of DEGs

The expression profile was preprocessed by Oligo package. After processed, DEGs between groups were screened out using the limma R package; *p*-value between gene expressions was determined using the *T* test and adjusted using Benjamini–Hochberg (BH) method. Those genes with the cut-off criteria of *p*-value < 0.05 and |log2fold change (FC)|> 0.58 were considered as DEGs.

## Construction of TFs–mRNA network

We use TRRUST database-identified TFs, we find the TFs–mRNA functional relation by Spearman correlation, the screen condition is *p* value < 0.01, cor > 0.9. The visualized network was generated by Cytoscape software.

## GO and KEGG enrichment analysis

Gene Ontology (GO) and Kyoto Encyclopedia of Genes and Genomes (KEGG) analysis of DEGs was performed with metascape (https://metascape.org/). GO analysis mainly reflects three aspects of gene information: biological process (BP), molecular function (MF), and cellular component (CC).

## PPI network construction

The protein–protein interaction (PPI) network of DEGs were constructed using the Search Tool for the Retrieval of Interacting Genes/Proteins (STRING, https://string-db.org/),demonstrate the interaction relationships between DEGs. The whole network was further imported into Cytoscape software (https://cytoscape.org/), an open-source platform for visualizing complex networks and analyzed with its MCODE.

## Immune cell composition

Cell-type enrichment scores were computed by running xCell on the full TPM gene expression matrix. In this study, we use the xCell to analyze the different immune cell subtypes between unstable plaque and stable plaque.

## Results

### Identification of differentially expressed mRNA in unstable carotid plaque (identification of DEGs)

**Fig. 1:**
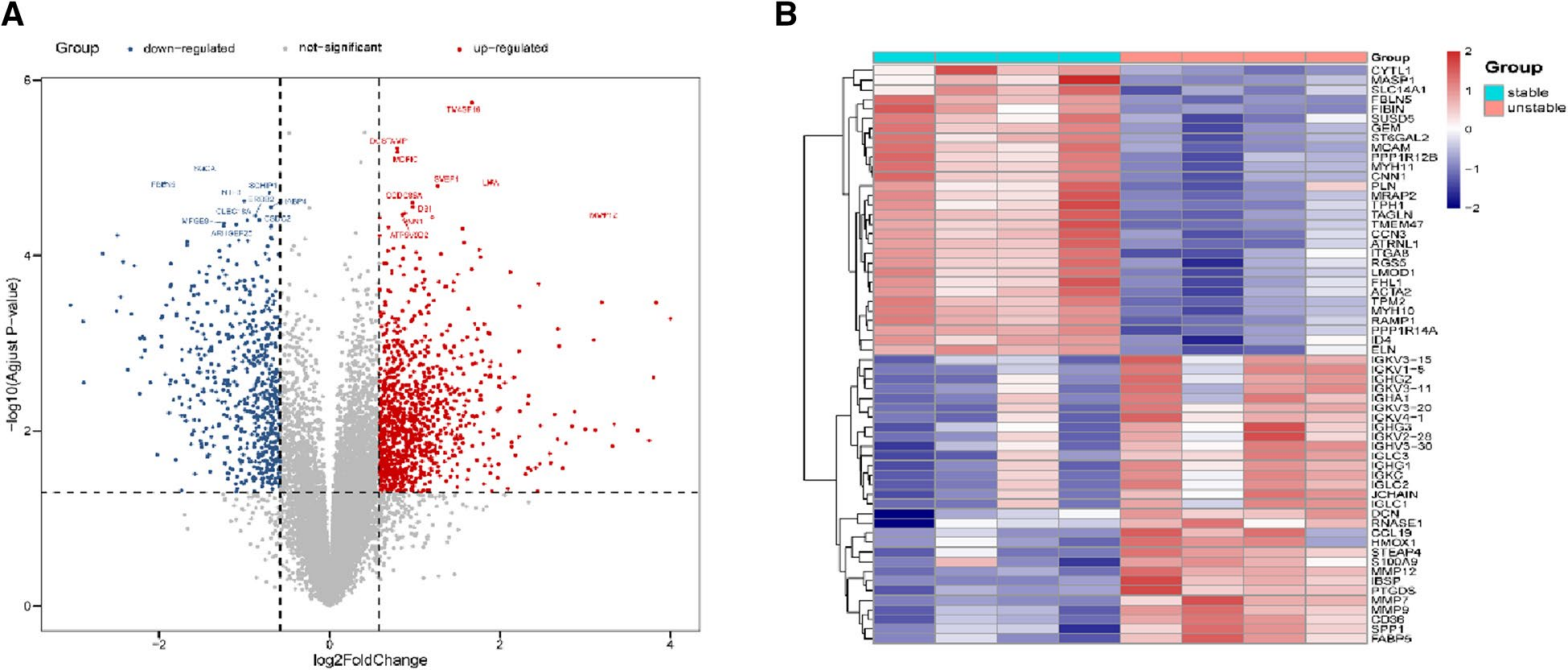
Identification of DEGs in controls and unstable carotid plaque. **A** Volcano plots showing the top 10 DEGs, the red dots represent up-regulated and the blue dots represent down-regulated mRNAs. **B** Heatmap illustrating the top differentially expressed genes (DEGs), red represents relatively high and blue represents relatively low

Raw data of the high-throughput sequencing datasets were downloaded from GEO database and then further processed with Limmaffy in R package. As a result, compared with stable plaque, there have differentially expressed genes (DEGs), there are 1034 up-regulated and 592 down-regulated genes in unstable plaque of atherosclerosis (Supplementary Excel1).According to adjust *p*-value, TM4SF19, DCSTAMP, MDFIC, LIPA, SVEP1, CCDC88A, DBI, VNN1, MMP12, ATP6V0D2 were ranked among the top 10 up-regulated DEGs (Table1, Fig. 1A); and viceSGCA, FBLN5, SCHIP1, NTF3, HABP4, ERBB2, CSDC2, CLEC18A, MFGE8, ARHGEF25-1 had the most significant changes in down-regulated DEGs (Table1, Fig. 1B).

**Table1:** Top 10 DEG mRNA between unstable plaque and stable plaque

Top 10 mRNAs	Up-regulation				Down-regulation		
		Log FC	Adjusted P value			Log FC	Adjusted P value
1	TM4SF19	1.667968	0.024586	SGCA		− 1.47932	0.027648
2	DCSTAMP	0.789685	0.024586	FBLN5		− 1.93589	0.030007
3	MDFIC	0.793604	0.024586	SCHIP1		− 0.70644	0.030278
4	LIPA	1.889806	0.030007	NTF3		− 1.00411	0.030278
5	SVEP1	1.271239	0.030007	HABP4		− 0.68798	0.030278
6	CCDC88A	0.976038	0.030278	ERBB2		− 0.87112	0.030278
7	DBI	0.981414	0.030278	CSDC2		− 0.82249	0.030278
8	VNN1	0.883017	0.030278	CLEC18A		− 0.96871	0.030278
9	MMP12	3.227392	0.030278	MFGE8		− 1.23571	0.030278
10	ATP6V0D2	0.855449	0.030278	ARHGEF25		− 1.09899	0.030278

### GO and KEGG analysis of DEGs

**Fig. 2:**
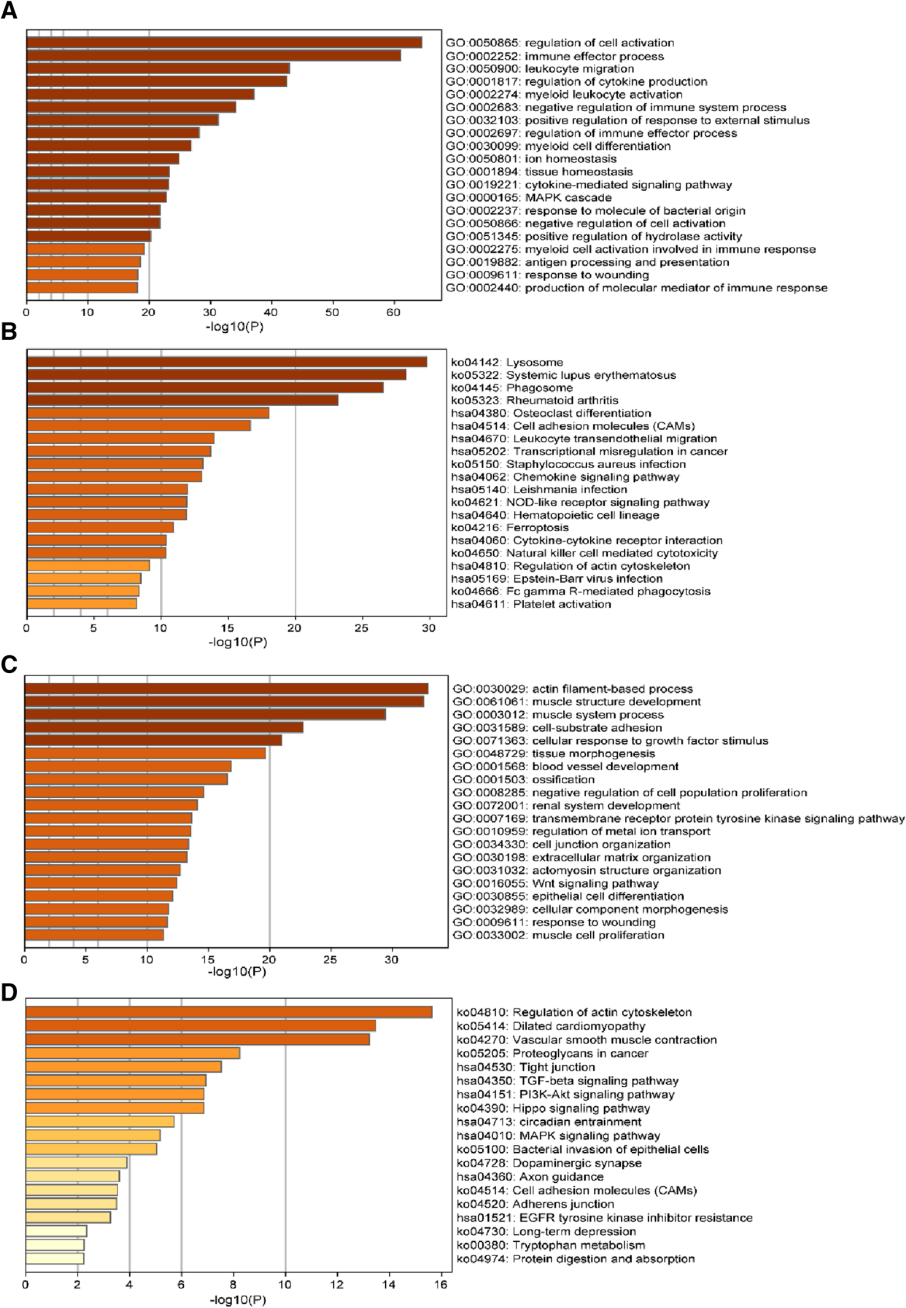
GO and KEGG pathway analysis of DEGs between carotid unstable plaque and stable plaque. **a**, **c** Show the major GO Biological process analysis of all up-regulated and down-regulated mRNAs. **b**, **d** The most KEGG signaling pathway enrichment of all up-regulated and down-regulated mRNAs

GO and KEGG pathway analyses were also performed on all the DEGs to fully explore the biological role of all the DEGs, which include all 1034 and 592 target genes. Among the up-regulated DEGs, the GO biological processes were involved in regulation of cell activation, immune effector process, leukocyte migration, regulation of cytokine production, myeloid cell activation involved in immune response. The KEGG pathway were involved the ‘lysosome’ pathway, chemokine signaling pathway, leukocyte trans-endothelial migration, Fc gamma R-mediated phagocytosis, platelet activation. Among the down-regulated genes, the GO biological processes were related to actin filament-based process, muscle structure development, muscle system process, cell-substrate adhesion; the KEGG pathway were related to vascular smooth muscle contraction and TGF-beta signaling pathway, PI3K–Akt signaling pathway, MAPK signaling pathway, adherens junction (Fig. 2A–D).

### Prediction of upstream TFs and downstream TFs target genes of DEGs

**Fig. 3:**
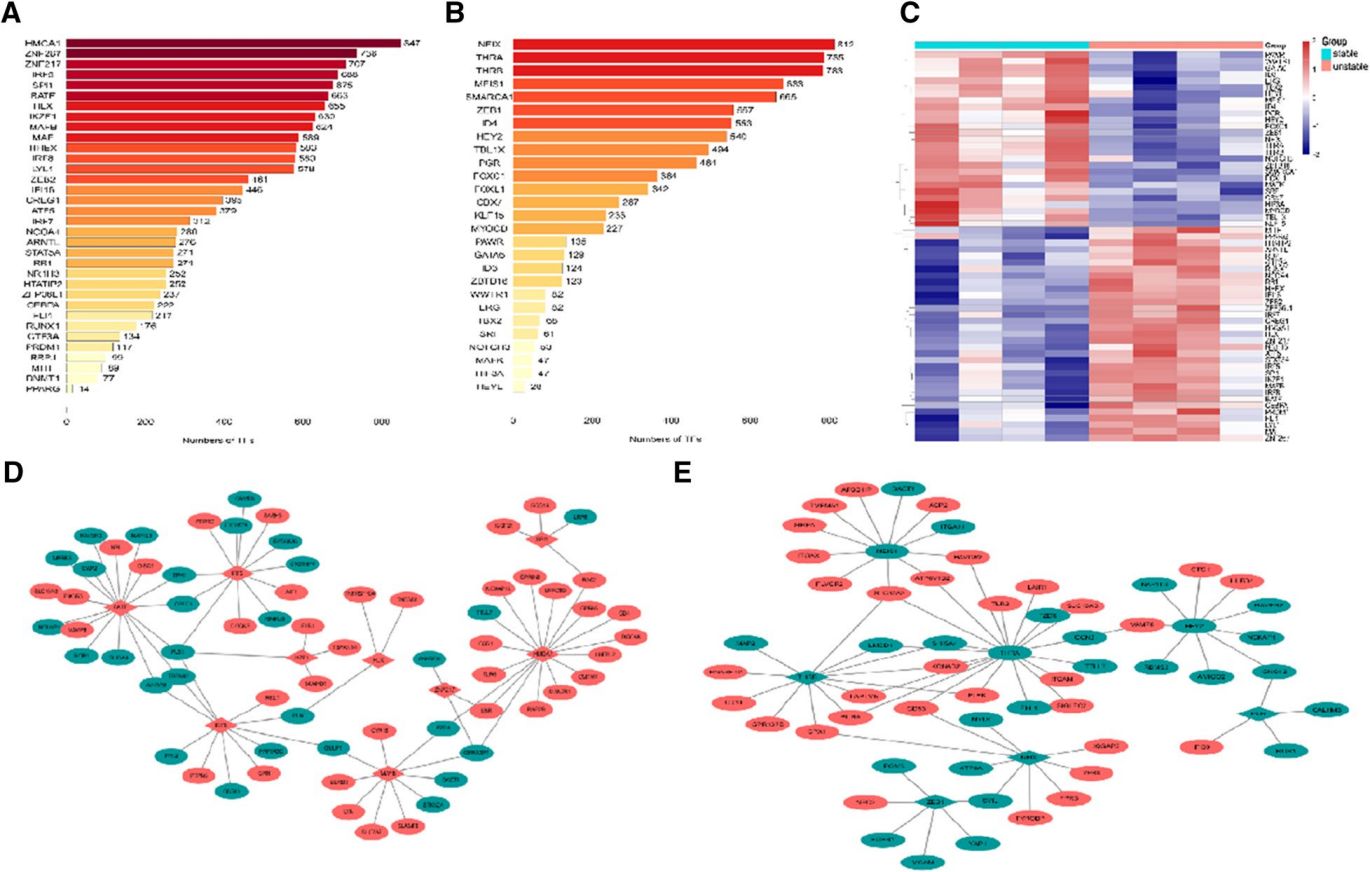
Analysis upstream and downstream TFs of DEGs between unstable carotid plaque and stable plaque. **A** Transcription factors of all up-regulated mRNAs. **B** Transcription factors of the all down-regulated mRNAs. **C** The heatmap of upstream and downstream TFs. TFs regulates the network diagram of mRNA. The red diamond in the center represents the up-regulated TFs–mRNA, the surrounding red ellipses represent the up-regulated mRNA, and the green ellipses represent the down-regulated mRNA **D**; the green diamond in the center represents the down-regulated TFs–mRNA, the surrounding red ellipses represent the up-regulated mRNA, and the green ellipses represent the down-regulated **E**, which show top 50 TFs–mRNAs pairs

I pulled 62 transcription factors from the Trust database. Indeed, 34 up-regulated and 28 down-regulated TFs were identified (Fig. 3A, B), and is presented as Heatmap (Fig. 3C). 13,532 TF–mRNA pairs were found in 34 up-regulated TFs, 8748 TF–mRNA pairs were found in 27 down-regulated TFs. Transcriptional regulatory networks indicated *HMGA1* as the top-ranked regulator of up-regulated TFs, which promotes epithelial-to-mesenchymal transition in cancer and associated with PAH [10]; NFIX is the top-ranked regulator of down-regulated TFs(Fig. 3A). In order to display the regulatory network directly, we continued to construct the complex TFs–mRNA network by TFsNET, We show the top 50 TFs–mRNA pairs network (Fig. 3D, E).

### GO analysis of TFs

**Fig. 4:**
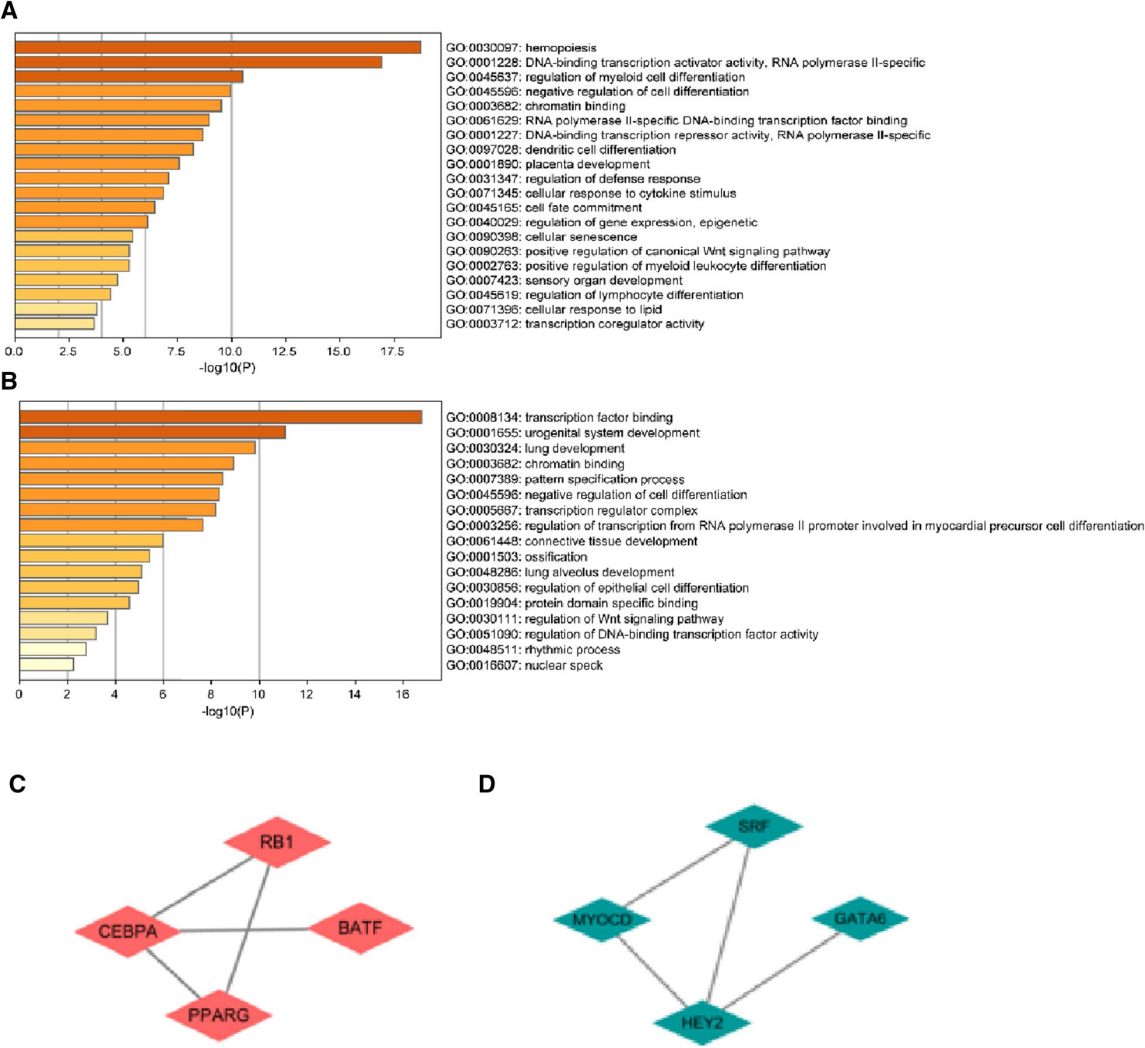
GO analysis of TFs. **a** The major GO enrichment of up-regulated TFs, **b** the major GO enrichment of down-regulated TFs. **c** Upregulated TFs of PPI network; **d** down-regulated TFs of PPI network.TF, transcription factor

The up-regulated TFs were mainly enriched in positive regulation of myeloid leukocyte differentiation, and the down-regulated TFs were mainly enriched in connective tissue development (Fig. 4A, B). TF protein interaction network could also be obtained based on metascape database, in which macrophage differentiation and myeloid cell differentiation were identified, according to this, wo select 4 TFs(RB1, CEBPA, PPARG, BATF)for further investigated from all up-regulated TFs (Fig. 4C); TFs protein interaction network could also be obtained based on metascape database, in which cardiac vascular smooth muscle cell differentiation was identified, 4 down-regulated TFs (SRF, MYOCD, HEY2, GATA6) were selected from all TFs (Fig. 4D).

### Immune cell composition in the carotid unstable plaque and stable plaque

**Fig. 5:**
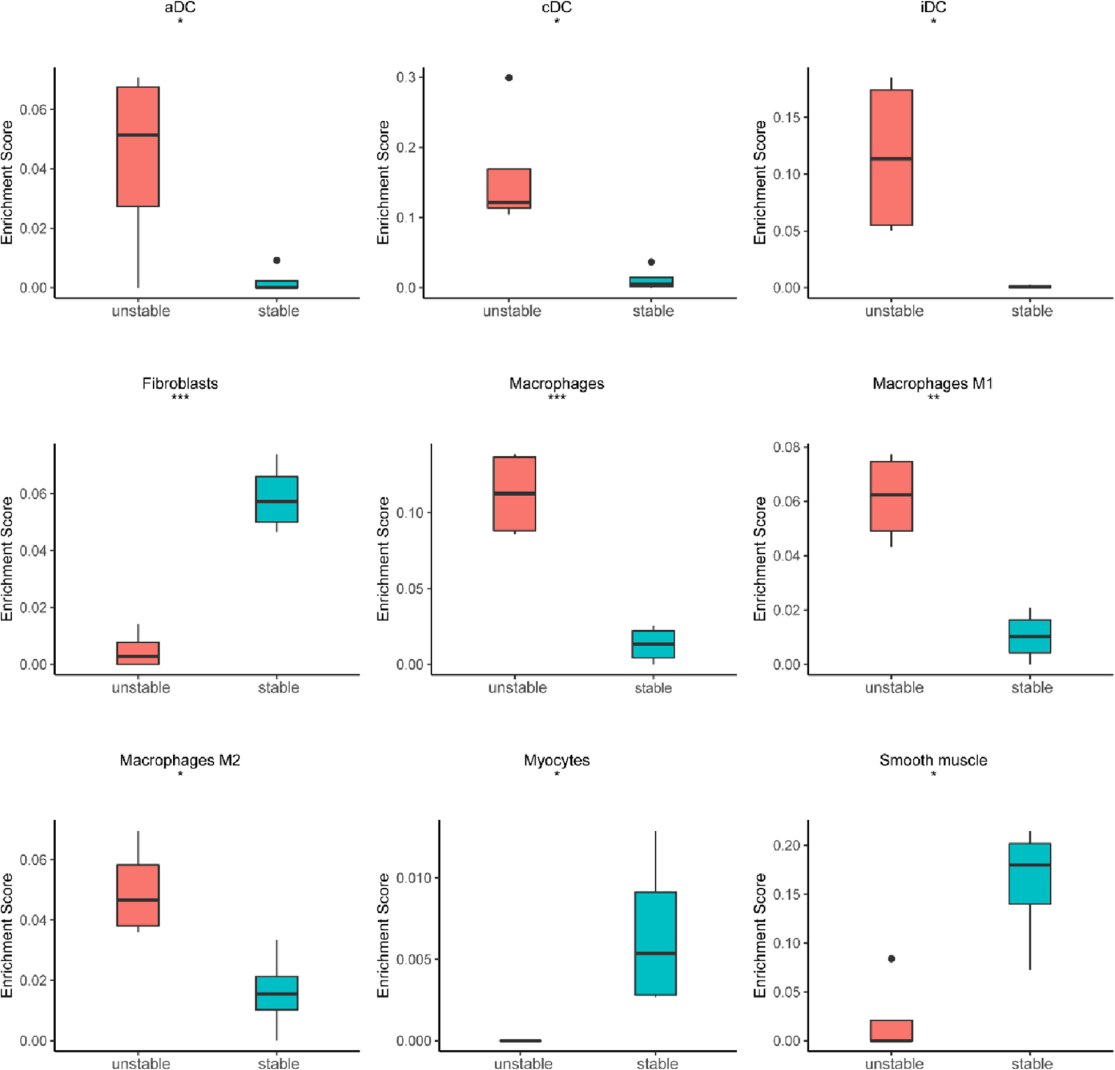
Immune cell composition generated by xCell-inferred enrichment score of cell types across unstable plaque and stable plaque. *p < 0.05; **p < 0.01; ***p < 0.001 by Kruskal–Wallis test

The result of immune cell composition of DEGs showed that the up-regulated DEGs were activated in the DC cell and macrophages, which enrichment score were significant differences in the unstable carotid plaque (*p* < 0.05), down-regulated DEGs were activated in the fibroblasts, myocytes, smooth muscle, which enrichment score were significant differences (*p* < 0.05) in the stable carotid plaque (Fig. 5).

## Discussion

The role of inflammation in the formation, vulnerability and rupture of atherosclerotic plaque has received increasing attention. Macrophages are key instigators of atherosclerotic inflammation, but are also critical for the regression phase of the inflammatory process, and their contribution depends on their activation state. Rewiring the transcriptional state of macrophages is an attractive therapeutic strategy for CVD to prevent excessive inflammation [11]. Through bioinformatics analysis, we screened out hub genes that affect the stability of atherosclerotic plaques, as well as related biological processes and immune cells.

After reanalyzing mRNA high-throughput dataset of carotid plaque, compare to the stable plaque, we found that these upregulated mRNAs were enrichment in biological process including immune effector process and myeloid effector activation. This result is consistent with some studies, their result suggest that innate and adaptive immune dysregulation play an important role at the atherosclerotic site in patients with recent CV events [6, 12]. However, in a recent work of screening key TFs in regulating plaque stability with mRNA expression profiles, According to the up-regulated mRNA and down-regulated mRNA to predict upstream TFs and downstream TFs targets.

We select 4 up-regulated hub genes (*RB1/CEBPA/PPARG/BATF*) by gene expression signature in unstable carotid plaque. Among them, the retinoblastoma susceptibility gene (*RB1*) was the first tumor suppressor gene to be molecularly defined. Product RB1(pRB) is a chromatin-associated protein that limits the transcription of cell cycle genes, primarily via regulation of the E2F transcription factor [13]. It is involved in the development of many tumor diseases, for example, human glioblastoma [14], Human Colon Cancer [15], small cell lung cancer [16], and so on. However, it is not clear in the mechanism of cardiovascular disease. In 2019, Prestel, M et al. founding that allele-specific transcriptional regulation of HDAC9 via E2F3 and RB1 as a major mechanism mediating vascular risk at rs2107595 [17]; but Yu Cao et al. another findings reveals that endothelial RB positively impacts arterial function by supporting vasoprotective endothelial DHFR/NO pathway activity, leading to reduced abdominal aortic aneurysm (AAA) development [18], their results are inconsistent. In the results of our analysis, RB1 expression was increased in unstable plaques, Therefore, further animal experiments are needed to confirm this.

*CEBPA* as a myeloid transcription factor, its mutations play an important role in the pathogenesis of hematological tumors [19, 20]. But it is less well studied in cardiovascular disease and in promoting atherosclerosis. A research showed that C/EBP transcription factors mediated epicardial activation during heart development and injury, disruption of C/EBP signaling in the adult epicardium reduced injury-induced neutrophil infiltration and improved cardiac function [21]. However, no studies have shown the mechanism of its role in the progression of atherosclerotic plaque. Our analysis of the results showed that he was up-regulated in unstable plaques, At the same time, a single-cell sequencing database analysis showed that (https://panglaodb.se/index.html) *CEBPA* was highly expressed in macrophages(Additional file 1: figure S1). Therefore, we infer that it plays an important role in the development of atherosclerosis.

Another transcription factor, The peroxisome proliferator-activated receptor gamma gene (*PPARG*), encodes a member of the peroxisome-activated receptor subfamily of nuclear receptors. PPARG-gamma has been implicated in the pathology of numerous diseases including obesity, diabetes, atherosclerosis and cancer [22], and also plays a key role in subcellular metabolism of arterial wall macrophage foam cells [23]. Genetic variation in *PPARG* has been associated with metabolic and cardiovascular end points. Khalid Z Al-Shali et al. results suggested that PPARG c.1431 T allele had greater total carotid plaque volume (TPV) than others, with no between-genotype difference in IMT. The findings show an association between *PPARG* genotypes and carotid arterial phenotypes [24]. Our bioinformatics analysis suggest that it was up-regulated in unstable plaques, Therefore, the mechanism of its effect on vascular plaque stability needs to be explored in further experiments.

Transcription factor *BATF* as a key transcription factor, regulation of Treg differentiation in non-lymphoid cells [25], which most are used to regulate T cells in viral and bacterial infections and in tumor immunity disease [26, 27], very little research has been done on cardiovascular disease. One studies have shown that after in vitro and in vivo injection of endotoxin and LPS, six candidate genes (*BATF*, BID, C3aR1, IL1RN, SEC61B and SLC43A3) were identified to be associated with inflammation and atherosclerosis [28]. Another study demonstrate that the *BATF* and BATF3 are critical regulators of T effector functions, thus making them attractive targets for therapeutic interventions in heart allograft [29]. Our xCell results showed that the number of macrophages in unstable plates increased significantly, indicating that the number and function of macrophages play a very important role in the progression of plaques, above studies indicate that *BATF* is mainly involved in the progression of cardiovascular disease as a regulatory factor of T cells, a single-cell sequencing database analysis showed that (https://panglaodb.se/index.html) *BATF* was also highly expressed in macrophages, so, it may be involved in regulating plaque instability or rupture.

*SRF, MYCOD, HEY2, GATA6* are as transcription factors, our results showed that they were down-regulated in unstable plaques, whereas they were up-regulated in stable plaques. Its xCell results turned out to be fibroblasts and muscle cells (*p* < 0.001). These transcription factors are involved in the molecular regulation of heart development and disease. For example, SRF、MYOCD are related to the development of cardiac vascular smooth muscle and molecular regulation of diseases [30–32], Mutations in HEY2 cause Brugada syndrome, a rare heart disease [33, 34]. Fibroblast GATA6 promotes myocardial adaptation to pressure overload by enhancing cardiac angiogenesis [35].

TFs protein interaction network could also be obtained based on metascape database, in which macrophage differentiation and myeloid cell differentiation were identified. This is consistent with immune cell composition result. Single-cell sequencing of some atherosclerotic plaques found that T cells and macrophages dominate the atherosclerotic plaque immune landscape [6].

## Conclusion

In conclusion, findings in the current study demonstrated that the vulnerability or unstable atherosclerotic plaques might be the result of imbalance between macrophages and fibrosis. Crosstalk between macrophages and SMCs may be involved in this process. Specifically, up-regulated *RB1, CEBPA, PPARG, BATF*, and down-regulated *SRF, MYCOD, HEY2, GATA6* performed important promotional effects for the rupture of atherosclerotic plaques. These findings provide a theoretical basis for the future study of specific mechanisms of atherosclerotic rupture.

## Supplementary Information

**Additional file 1: Figure S1. CEBPA** was highly expressed in macrophages. This analysis comes from https://panglaodb.se/index.html.

**Additional file 2: Excel S1.** All the differentially expressed genes between unstable and stable plaque of atherosclerosis.

## Data Availability

All data generated or analyzed during this study are included in this published article.
