# Supplementary material for: The underlying molecular mechanisms and biomarkers of plaque vulnerability based on bioinformatics analysis
Source: Eur J Med Res. 2022 Oct 27;27:212. doi: 10.1186/s40001-022-00840-7 (PMC9615401; doi:10.1186/s40001-022-00840-7)
Supplement: Supplementary file 1 — Additional file 1: Figure S1. CEBPA was highly expressed in macrophages. This analysis comes from https://panglaodb.se/index.html. [file 40001_2022_840_MOESM1_ESM.docx]

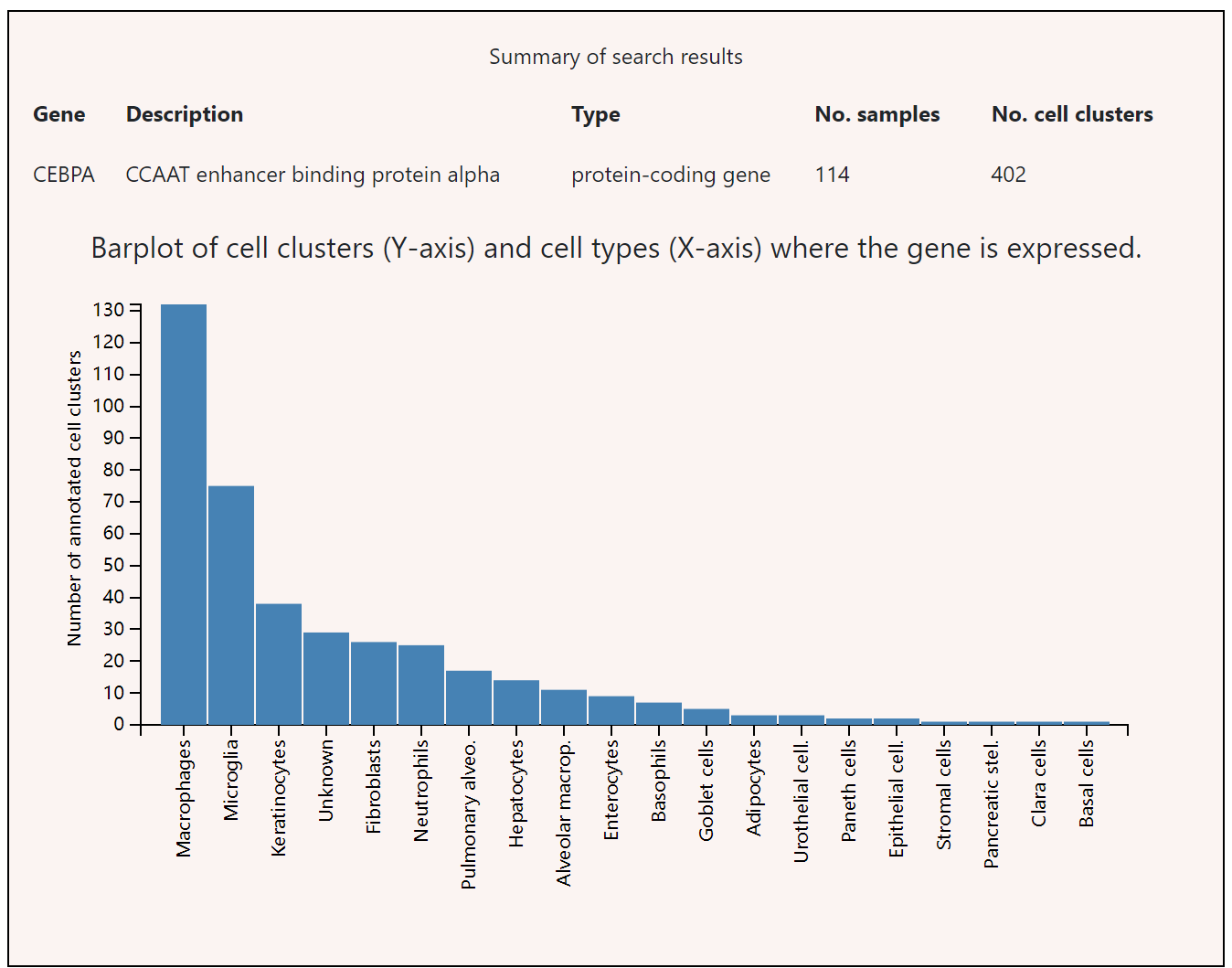


Supplementary figure1：*CEBPA* was highly expressed in macrophages

This analysis comes from https://panglaodb.se/index.html
